# Complete genome of *Aedes japonicus* narnavirus from wild caught mosquitoes collected in Ohio, USA.

**DOI:** 10.17912/micropub.biology.000909

**Published:** 2023-09-05

**Authors:** Ferdinand Nanfack-Minkeu, Alexander Delong, William Morgan

**Affiliations:** 1 Department of Biology , College of Wooster, Wooster, Ohio, United States; 2 Biochemistry & Molecular Biology Program, College of Wooster, Wooster, Ohio, United States; 3 Department of Biology, College of Wooster, Wooster, Ohio, United States

## Abstract

Narnaviruses infect several genera of mosquitoes including *Culex* and *Aedes* . The narnavirus genome is a positive, single stranded RNA encoding an RNA-dependent RNA polymerase gene. The partial genome of a narnavirus identified in wild *Aedes japonicus* mosquitoes collected in Wooster, Ohio, USA was obtained using metagenomic analyses. Rapid amplification of 5’-cDNA ends (RACE) and Sanger sequencing were used to obtain the remaining genomic sequence of this strain. The complete genome is composed of 3153 nucleotides and has 98.4% and 99.1% nucleotide sequence identity with *Aedes japonicus* narnavirus genomes identified in Netherlands and Japan.

**Figure 1. Genomic and phylogenetic analysis of the Wooster AJNV isolate:**
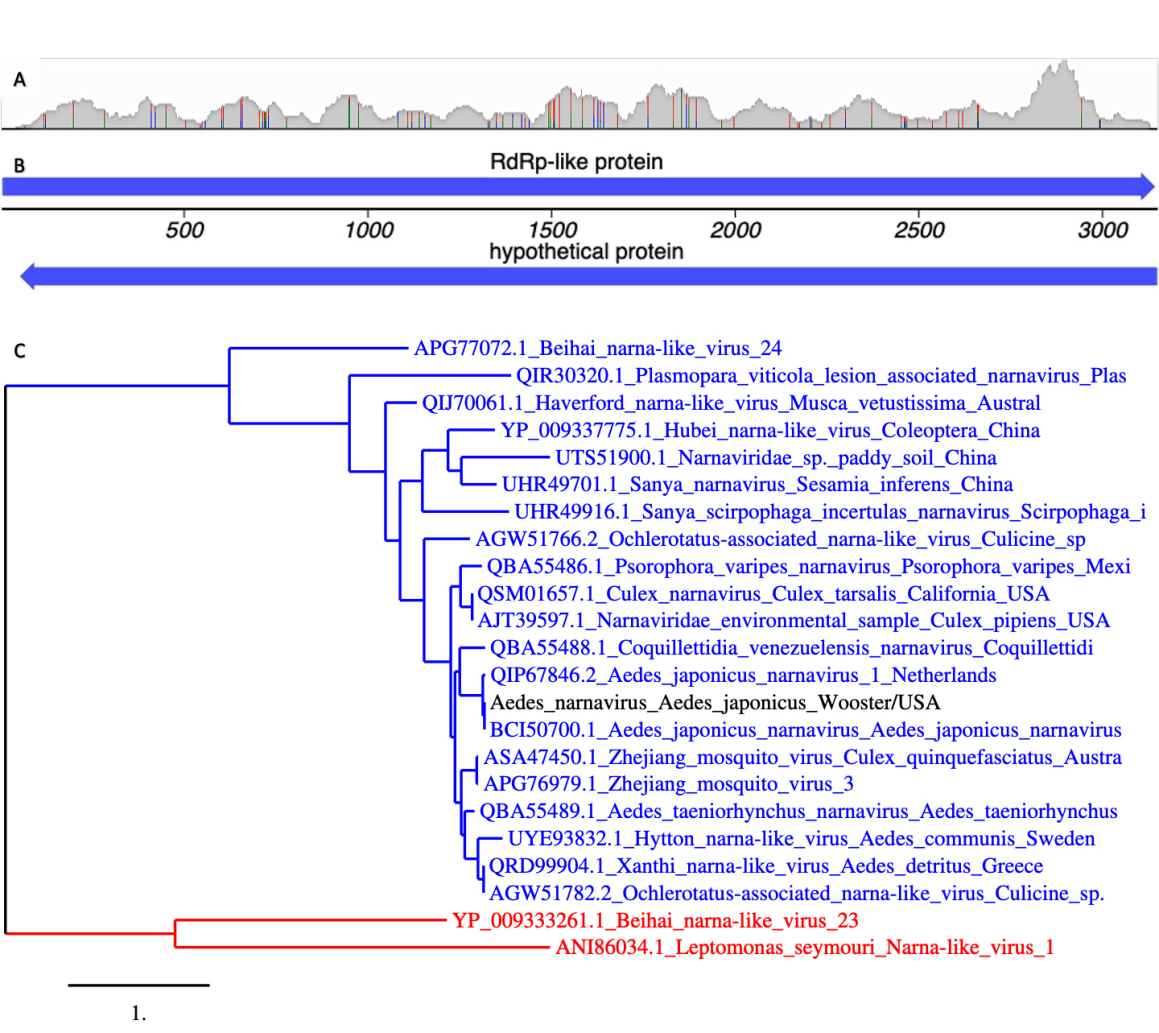
(A) Read depth coverage. Depth of coverage (0-35000) of Illumina reads aligning to the LC567882.1 reference sequence. The 86 sites exhibiting variation between reads are indicated by colored vertical lines. (B) Coding potential. The full-length genomic sequence can encode an RNA-dependent RNA polymerase (RdRp)-like protein on its forward strand and a hypothetical protein on its reverse strand. (C) Phylogenetic tree. Evolutionary relationship of the Wooster isolate (black) and representative alphanarnaviruses (blue) and betanarnaviruses (red) based on predicted RdRp amino acid sequences. Scale bar at bottom indicates branch length measured in nucleotide substitutions per site.

## Description

The virome of mosquitoes can be grouped into arboviruses and Insect specific viruses (ISVs). Arboviruses, including West Nile virus, Lacrosse virus and Eastern encephalitis equine virus, are pathogenic to vertebrates. Unlike arboviruses, ISVs, including *Aedes japonicus* narnavirus (AJNV), infect and replicate only in insects and are non-pathogenic to vertebrates (Bolling et al., 2015; Nanfack Minkeu and Vernick, 2018; Nanfack-Minkeu et al., 2019; Dinan et al., 2020) . AJNV belongs to the Narnaviridae family, and its genome is a single molecule of non-encapsidated positive-strand RNA coding for an RNA-dependent RNA polymerase gene. Many members of the alphanarnaviral subgroup also possess a reverse ORF able to encode a ~1000 amino acid protein of unknown function, while betanarnaviruses do not (Dinan et al., 2020) . Narnaviruses have been detected in diverse fungal, plant and insect species with genomic variation observed between hosts and locations. The impact of the genomic variations is unknown in several hosts. A metagenomic analysis of *Aedes japonicus* mosquitoes collected in 2021 (Nanfack-Minkeu et al., 2023) and 2022 in Wooster, Ohio, USA, revealed a partial AJNV genome. Assembled nodes of the metagenomic sequence spanning approximately 3 kb aligned with previously sequenced AJNV genomes (LC567882.1, MK984721.1) (Abbo et al., 2020) , but approximately 200 bp was missing at the 5’ end ( Figure 1A ). The 5’- RACE and Sanger sequencing procedures were used to reveal a complete genome of 3153 nucleotides. This genome contains an exceptionally long, reverse ORF ( Figure 1B ) and encodes an RNA-dependent RNA polymerase highly similar to other alphanarnaviruses ( Figure 1C ). Remarkably, the AJNV genome of the Wooster USA isolate (accession number : OR258984) has 98.4% and 99.1% nucleotide sequence identity with AJNV genomes isolated from *Ae. japonicus* collected in Netherlands (MK984721.2) and maintained in Japan (LC567882.1), respectively, suggesting a highly conserved virus between different countries (Abbo et al., 2020; Faizah et al., 2020) .

## Methods

Mosquito collection: Wild *Aedes japonicus* mosquitoes were collected using gravid traps in Wooster, Ohio between June and September 2021 and 2022. The details of collection and identification (Extended Data) were previously described (Nanfack-Minkeu et al., 2023) .

Mosquito RNA isolation: The pools (25 to 30) of *Ae. japonicus* mosquitoes were crushed with 500µL of Trizol using a pestle. Then, 200 μL of chloroform was added, and the mixture was shaken by hand to mix. The sample was then centrifuged at 16,000 RPM for 15 minutes at 4°C. A phaselock gel heavy 2 mL tube (PLG) (Quantabio, USA) was pre-centrifuged at 12,000 RPM for 30 seconds at room temperature, and then 200 μL chloroform was added into the PLG tube. Once centrifugation of the mosquito sample was complete, the top aqueous layer was transferred into the PLG tube. The PLG tube was then shaken by hand until the sample was mixed and then centrifuged at 12,000 RPM for 5 minutes at 4°C. After centrifugation, the top layer was then pipetted into a new 2 mL tube containing 250 μL isopropanol. RNA was then isolated following the manufacturer’s protocol (Invitrogen, USA). The pellet was resuspended in 40 μL of RNAse-free water. A NanoDrop spectrophotometer was used to assess the concentration and quality of RNA. RNA was stored at –80°C until deep sequencing.

Metagenomic analysis: Illumina deep sequencing was conducted by Medgenome (USA) using the Illumina TruSeq stranded total RNA library preparation and the NovaSeq (PE100/150) machine. Viral sequences from Medgenome were de novo assembled in Chan-Zuckerberg ID (formerly IDSeq, https://czid.org) using metagenomic next generation sequencing pipeline version 6.8 with default parameters composed of the following softwares: Trimmomatic, STAR, Genomic Short-read Nucleotide Alignment Program (GSNAP), Bowtie 2 and RAPSearch2 (Langmead and Salzberg, 2012; Zhao, Tang and Ye, 2012; Dobin et al., 2013; Wu et al., 2016) . Bowtie 2 alignments were visualized with the Integrated Genomic Viewer (v2.16.1) (Robinson et al., 2017) .

5’ Rapid amplification of cDNA ends (RACE): The 5′ RACE protocol was performed following the manufacturer’s instructions (New England Biolabs, MA, USA). After annealing the AJNV-specific primer AJNV-R3 with purified mosquito RNA, reverse transcription and template switching was performed with TSO-A (Table 1). This first strand cDNA was then used as a template to PCR amplify the 5’ end of the viral genome using Phusion high-fidelity DNA polymerase (NEB) with the AJNV-R3 and TSO-Aspec primers (Table 1). The PCR product was purified using an ExoSAP treatment (Thermo Fisher, USA) following the manufacturer's instructions. Sanger sequencing of the PCR product was performed by Eurofins Genomics (Louisville, KY USA) using the TSO_Aspec or AJNV-R3 primer. This sequence was aligned with the MK984721.1 reference sequence and the metagenomic nodes (contigs) using CodonCode Aligner (v8.0.1) to generate a full-length consensus sequence. MacVector (v18.2.5) was used to identify open reading frames and predicted amino acid sequences. The Basic Local Alignment Search Tool (Blastn) was used to align the consensus nucleotide sequence with the most closely related genomes in NCBI's Nucleotide (nr/nt) database to determine percent nucleotide identity (Altschul et al., 1990) .

Phylogenetic analysis: Phylogenetic analysis was performed using the standard pipeline at phylogeny.fr (Dereeper et al., 2008) . Briefly, the predicted RdRp amino acid sequence of the Wooster, OH isolate was aligned with MUSCLE to representative alpharnavirus RdRp sequences, with betanarnavirus RdRp sequences as the outgroup (see Figure 1C ; Edgar, 2004; Dinan et al., 2020). Following curation with GBlocks, the phylogenetic tree was constructed from the aligned sequences using the default settings of PhyML and visualized with TreeDyn (Anisimova and Gascuel, 2006; Chevenet et al., 2006; Dereeper et al., 2008, 2010; Nguyen et al., 2015) .

Table 1 : List of primers

**Table:** 

Description	Name	Sequence
Template switching oligo (NEB)	TSO_A	GCTAATCATTGCAAGCAGTGGTATCAACGCAGAGTACATrGrGrG
TSO-A-specific (NEB)	TSO_Aspec	CATTGCAAGCAGTGGTATCAAC
AJNV-specific reverse	AJNV_R3	GGCGTACTCCTTGACTTGCT

## Extended Data

Description: Extended Data : Coordinates of collection sites . Resource Type: Image. DOI: 10.22002/e8ge1-rs265

## References

[R1] Abbo SR, Visser TM, Wang H, Göertz GP, Fros JJ, Abma-Henkens MHC, Geertsema C, Vogels CBF, Koopmans MPG, Reusken CBEM, Hall-Mendelin S, Hall RA, van Oers MM, Koenraadt CJM, Pijlman GP (2020). The invasive Asian bush mosquito Aedes japonicus found in the Netherlands can experimentally transmit Zika virus and Usutu virus.. PLoS Negl Trop Dis.

[R2] Altschul SF, Gish W, Miller W, Myers EW, Lipman DJ (1990). Basic local alignment search tool.. J Mol Biol.

[R3] Anisimova M, Gascuel O (2006). Approximate likelihood-ratio test for branches: A fast, accurate, and powerful alternative.. Syst Biol.

[R4] Bolling BG, Weaver SC, Tesh RB, Vasilakis N (2015). Insect-Specific Virus Discovery: Significance for the Arbovirus Community.. Viruses.

[R5] Chevenet F, Brun C, Bañuls AL, Jacq B, Christen R (2006). TreeDyn: towards dynamic graphics and annotations for analyses of trees.. BMC Bioinformatics.

[R6] Dereeper A, Guignon V, Blanc G, Audic S, Buffet S, Chevenet F, Dufayard JF, Guindon S, Lefort V, Lescot M, Claverie JM, Gascuel O (2008). Phylogeny.fr: robust phylogenetic analysis for the non-specialist.. Nucleic Acids Res.

[R7] Dereeper A, Audic S, Claverie JM, Blanc G (2010). BLAST-EXPLORER helps you building datasets for phylogenetic analysis.. BMC Evol Biol.

[R8] Dinan AM, Lukhovitskaya NI, Olendraite I, Firth AE (2020). A case for a negative-strand coding sequence in a group of positive-sense RNA viruses.. Virus Evol.

[R9] Dobin A, Davis CA, Schlesinger F, Drenkow J, Zaleski C, Jha S, Batut P, Chaisson M, Gingeras TR (2012). STAR: ultrafast universal RNA-seq aligner.. Bioinformatics.

[R10] Edgar RC (2004). MUSCLE: multiple sequence alignment with high accuracy and high throughput.. Nucleic Acids Res.

[R11] Langmead B, Salzberg SL (2012). Fast gapped-read alignment with Bowtie 2.. Nat Methods.

[R12] Nanfack Minkeu F, Vernick KD (2018). A Systematic Review of the Natural Virome of Anopheles Mosquitoes.. Viruses.

[R13] Nanfack-Minkeu F, Mitri C, Bischoff E, Belda E, Casademont I, Vernick KD (2019). Interaction of RNA viruses of the natural virome with the African malaria vector, Anopheles coluzzii.. Sci Rep.

[R14] Nanfack-Minkeu F, Delong A, Luri M, Poelstra JW (2023). Invasive Aedes japonicus Mosquitoes Dominate the Aedes Fauna Collected with Gravid Traps in Wooster, Northeastern Ohio, USA.. Insects.

[R15] Nguyen LT, Schmidt HA, von Haeseler A, Minh BQ (2014). IQ-TREE: a fast and effective stochastic algorithm for estimating maximum-likelihood phylogenies.. Mol Biol Evol.

[R16] Robinson JT, Thorvaldsdóttir H, Wenger AM, Zehir A, Mesirov JP (2017). Variant Review with the Integrative Genomics Viewer.. Cancer Res.

[R17] Shimada J, Yamakawa H (1988). Sedimentation coefficients of DNA topoisomers: the helical wormlike chain.. Biopolymers.

[R18] Wu TD, Reeder J, Lawrence M, Becker G, Brauer MJ (2016). GMAP and GSNAP for Genomic Sequence Alignment: Enhancements to Speed, Accuracy, and Functionality.. Methods Mol Biol.

[R19] Zhao Y, Tang H, Ye Y (2011). RAPSearch2: a fast and memory-efficient protein similarity search tool for next-generation sequencing data.. Bioinformatics.

